# Moveable Kidney: A Cause of Hepatic Colic

**Published:** 1900-01-13

**Authors:** 


[1] Lancet, Jan. 6.

### MOVEABLE KIDNEY: A CAUSE OF HEPATIC COLIC.

In a paper by Dr. Maclagan[1] and Mr. Treves the very interesting fact is recorded that a moveable kidney may by its pressure on the bile duct so interfere with the emptying of the gall-bladder as to give rise to all the symptoms of hepatic colic. Hale White[2] has already recorded a case of jaundice caused by the pressure of a moveable kidney upon the bile ducts, in which the jaundiced condition was removed by fixing the displaced organ; and Dr. Cordier,[3] of Kansas, has related a case of moveable kidney in which the gall-bladder was distended and the patient was at times slightly jaundiced, the symptoms all disappearing after an operation for anchoring the kidney; but the cases now recorded seem more important, as they show how the recurrent pains and of other symptoms, including jaundice, which are generally regarded as characteristic of gall-stones, may be set up by that condition so common in women, a moveable kidney. The matter is one to be borne in mind, for when repeated attacks of jaundice occur and systematic search fails to demonstrate the passage of a stone, it is a reasonable thing to conclude, as was concluded in one of the cases recorded, that although the stone is large enough to enter the cystic duct, it is too large to get through; a condition which one need hardly say means mischief. The presence of a moveable kidney, however, is no doubt the explanation of a not inconsiderable number of these cases. It is only of late years that the various forms of enteroptosis, of which a moveable kidney is one, have been seriously considered as factors in the production of disease, using the word as meaning something more than inconvenience; but the more these displacements are studied the wider and more far-reaching do their results appear.

[1] Lancet, Jan. 6. [2] Brit. Med. Jour., vol. i., 1892. [3] Amer. Jour. of Obstetrics, 1895.

